# Dr. Albert Steuben Hotaling

**Published:** 1913-10

**Authors:** 


Dr. Albert Steuben Hotaling, P. & S. of Baltimore, 1894, died in Syracuse August 8, aged 40.

